# Predictive Factors of Response to Immunotherapy in Lymphomas: A Multicentre Clinical Data Warehouse Study (PRONOSTIM)

**DOI:** 10.3390/cancers15164028

**Published:** 2023-08-09

**Authors:** Marion Detroit, Mathis Collier, Nathanaël Beeker, Lise Willems, Justine Decroocq, Bénédicte Deau-Fischer, Marguerite Vignon, Rudy Birsen, Frederique Moufle, Clément Leclaire, Elisabeth Balladur, Paul Deschamps, Adrien Chauchet, Rui Batista, Samuel Limat, Jean-Marc Treluyer, Laure Ricard, Nicolas Stocker, Olivier Hermine, Sylvain Choquet, Véronique Morel, Carole Metz, Didier Bouscary, Marie Kroemer, Jérémie Zerbit

**Affiliations:** 1 Pharmacy Department, Pitié-Salpêtrière Hospital, Greater Paris University Hospitals (AP-HP), Sorbonne University, 75013 Paris, France; marion.detroit@aphp.fr (M.D.); carole.metz@aphp.fr (C.M.); 2 Clinical Research Unit, Cochin Hospital, AP-HP, Centre Paris-Cité University, 75014 Paris, France; mathis.collier@aphp.fr (M.C.); nathanael.beeker@aphp.fr (N.B.); jean-marc.treluyer@aphp.fr (J.-M.T.); 3 Hematology Department, Cochin Hospital, AP-HP, Centre Paris-Cité University, 75014 Paris, France; lise.willems@aphp.fr (L.W.); justine.decroocq@aphp.fr (J.D.); benedicte.deau-fischer@aphp.fr (B.D.-F.); marguerite.vignon@aphp.fr (M.V.); rudy.birsen@aphp.fr (R.B.); didier.bouscary@aphp.fr (D.B.); 4 Adult Department, Hospital at Home, AP-HP, Centre Paris-Cité University, 75014 Paris, France; frederique.moufle@aphp.fr (F.M.); clement.leclaire@aphp.fr (C.L.); elisabeth.balladur@aphp.fr (E.B.); 5 Hematology Oncology Department, André Mignot Hospital, 78157 Le Chesnay, France; paul.deschamps@aphp.fr; 6 Hematology Department, University Hospital of Besançon, 25000 Besançon, France; achauchet@chu-besancon.fr; 7 Pharmacy Department, Cochin Hospital, AP-HP, Centre Paris-Cité University, 75014 Paris, France; rui.batista@aphp.fr; 8 Pharmacy Department, University Hospital of Besançon, 25000 Besançon, France; slimat@chu-besancon.fr (S.L.); mkroemer@chu-besancon.fr (M.K.); 9 French National Institute of Health and Medical Research (INSERM), Etablissement Français du Sang Bourgogne Franche-Comte (EFS BFC), UMR1098, RIGHT, University of Bourgogne Franche-Comté, 25000 Besançon, France; 10 Regional Pharmacovigilance Center, Pharmacology Department, Cochin Hospital, AP-HP, Centre Paris-Cité University, 75014 Paris, France; 11 Hematology Department, Saint Antoine Hospital, AP-HP, INSERM UMRs 938, Sorbonne University, 75012 Paris, France; laure.ricard@aphp.fr (L.R.); nicolas.stocker@aphp.fr (N.S.); 12 Hematology Department, Necker Hospital, AP-HP, Centre Paris-Cité University, 75015 Paris, France; olivier.herminie@aphp.fr; 13 Hematology Department, Pitié-Salpêtrière Hospital, AP-HP, Sorbonne University, 75013 Paris, France; sylvain.choquet@aphp.fr (S.C.); veronique.morel@aphp.fr (V.M.); 14 Cancer Treatment Unit, Pharmacy Department, Hospital at Home, AP-HP, Centre Paris-Cité University, 75014 Paris, France

**Keywords:** clinical data warehouse, real-world data, predictive factors of immunotherapy, lymphoma

## Abstract

**Simple Summary:**

Immunotherapy is increasingly used in lymphoma strategy. Risk-adapted therapeutical management and set-up scores to -stratify the most vulnerable patients by risk are becoming major concerns. With the continuing upward trend of real-world data usage in addition to clinical trial data, it is possible to test the feasibility of using data from clinical data warehouses (CDWs) to identify new predictive factors for response or toxicity to immunotherapy. Based on a large set of biological and clinical factors, our results confirm already known predictors factors of CAR T (chimeric antigen receptor T) cells: age, elevated lactate dehydrogenase, and C-Reactive Protein at the time of infusion. Additionally male gender, low hemoglobin, and hypo- or hyperkalemia are demonstrated to be predictive factors for progression after CAR T cell therapy. Thus, the attractiveness of CDW for generating data by building ever larger cohorts is proven, enabling significant results to be obtained in line with those previously described in the literature.

**Abstract:**

Immunotherapy (IT) is a major therapeutic strategy for lymphoma, significantly improving patient prognosis. IT remains ineffective for a significant number of patients, however, and exposes them to specific toxicities. The identification predictive factors around efficacy and toxicity would allow better targeting of patients with a higher ratio of benefit to risk. PRONOSTIM is a multicenter and retrospective study using the Clinical Data Warehouse (CDW) of the Greater Paris University Hospitals network. Adult patients with Hodgkin lymphoma or diffuse large-cell B lymphoma treated with immune checkpoint inhibitors or CAR T (Chimeric antigen receptor T) cells between 2017 and 2022 were included. Analysis of covariates influencing progression-free survival (PFS) or the occurrence of grade ≥3 toxicity was performed. In total, 249 patients were included. From this study, already known predictors for response or toxicity of CAR T cells such as age, elevated lactate dehydrogenase, and elevated C-Reactive Protein at the time of infusion were confirmed. In addition, male gender, low hemoglobin, and hypo- or hyperkalemia were demonstrated to be potential predictive factors for progression after CAR T cell therapy. These findings prove the attractiveness of CDW in generating real-world data, and show its essential contribution to identifying new predictors for decision support before starting IT.

## 1. Introduction

Over the past few years, the management of malignant lymphoma (ML) has been largely modified through the development of immunotherapy [1,2,3,4,5,6,7]. Impressive results obtained with CAR T (Chimeric antigen receptor T) cells in third line treatment or beyond have led to the initiation of clinical trials assessing their efficacy earlier in the therapeutic strategy. Recently, results from the ZUMA-7 and TRANSFORM trials have demonstrated their efficacy in second-line treatment for large B cell lymphoma [8,9]. 

Despite their efficacy, treatments with ICI (immune checkpoint inhibitor) and CAR T cells are associated with substantial toxicities. The main toxicities are immune-related adverse events (irAE) underlying their mechanism of action, cytokine release syndrome (CRS), and immune effector cell-associated neurotoxicity syndrome (ICANS) [10,11,12]. However, the efficacy of immunotherapies varies widely, with complete response rates in the order of 40–50% in clinical trials, demonstrating that less than half of patients with ML can benefit from them [7,13,14,15]. Therefore, the establishment of predictive factors for immunotherapy in the ML context in order to maximise therapeutic benefit and reduce toxicity is a challenging topic. 

A few studies have aimed to assess predictors of ICI efficacy and toxicity in patients with ML [16,17,18,19,20,21,22,23]. In contrast, most studies have sought to identify factors associated with the clinical efficacy and/or toxicity of CAR T cells. A low C-reactive protein (CRP) level on the day of CAR T cell infusion, associated with high absolute lymphocyte count at apheresis or low peak ferritin, was predictive of response to axicabtagene ciloleucel (axi-cel) according to Jacobson et al. [24]. Another study demonstrated that extra nodal infiltration of two or more sites and high lymphoma burden were risk factors for early progression after CAR T cell therapy when considering lactate dehydrogenase (LDH) level and total metabolic tumour volume [25]. Other studies, including the JULIET trial, have demonstrated that an elevated LDH level at baseline is associated with poorer overall survival (OS) [14,26,27]. Low tumour burden at baseline and low systemic inflammation, on the contrary, were found to be associated with durable axi-cel response [28]. Interestingly, beyond biological factors, the DESCAR-T registry demonstrated that the type of CAR T cells used was significantly associated with both progression-free survival (PFS) and OS [29]. Other studies have focused on the predictive factors of toxicities. A high EASIX (Endothelial Activation and Stress Index) score has been found to predict severe CRS and ICANS after CAR T cell therapy, setting up a relationship between LDH, creatinine, and platelet levels [30]. Additionally, in combination with ferritin and CRP value, the baseline EASIX score was associated with the onset of CRS and ICANS after axi-cel infusion [31]. The CAR-HEMATOTOX score was set up to stratify risk for hematological toxicity, infectious complications, and poor survival outcomes prior to CAR T cells infusion, and might be useful for risk-adapted management of these toxicities [32,33]. Other factors that might predict CAR T cell efficacy and/or toxicity are currently under investigation, such as the composition of the intestinal microbiome, tumour necrosis factor-alpha, acute kidney injury, high sensitivity troponin, and brain natriuretic peptide [34,35,36,37].

In the context of the establishment of massive healthcare databases, this study aimed to determine whether a clinical data warehouse or repository might be useful for predicting the toxicity and efficacy of immunotherapies used in ML. 

## 2. Materials and Methods

### 2.1. Study Design and Population

We conducted a real-world retrospective multicentre study (PRONOSTIM, NCT05450367). The study population included all consecutive patients with relapsed and refractory (R/R) diffuse large B cell lymphoma (DLBCL) and Hodgkin lymphoma (HL) treated with authorized anti-CD19 CAR T cell therapy and ICI (anti-Programmed cell Death protein 1 (anti-PD-1)) in 21 Greater Paris University Hospitals (AP-HP) from August 2017 to May 2022. Exclusion criteria were as follows: patients under 18 years old at the time of infusion, patients who opposed the collection of their personal data, and patients who received both CAR T cell and ICI therapy. Real-world data were retrospectively collected from medical records using the AP-HP clinical data warehouse (CDW). The study received approval from the Institutional Review Board (CES-22-03_PRONOSTIM, IRB00011591) of the scientific and ethical committee of the AP-HP. All subjects included in this study were informed about the reuse of their data for research, and subjects who objected to the reuse of their data were excluded from this study in accordance with French legislation. All procedures related to this work adhered to the ethical standards of the relevant national and institutional committees on human experimentation and with the Helsinki Declaration of 1975, as revised in 2008.

### 2.2. Data Collection

The AP-HP CDW contains available data on all inpatient visits for HL or DLBCL to 21 AP-HP hospitals, as follows: age, sex, body mass index (BMI), diagnosis and date, performance status, Ann Arbor stage, prognostic value (International Prognostic Score for HL [38] and International Prognostic Index for DLBCL [39]), comorbidities, number and type of prior lines of treatment before CAR T cells or ICI infusion, laboratory tests and microbiological results, occurrence, and AE grade. The sociodemographic and biological data used in this study were structured data obtained via automatic CDW extraction. Likewise, where applicable, the date of death (from any cause) was extracted from the Systems Medicalization Program. Comorbidities were extracted from International Classification of Diseases codes used during previous hospitalizations and before the ML diagnosis date of each patient, if available. ML characteristics, previous treatment details, outcomes, and toxicities (presence/absence and grade) were obtained by semi-automatic extraction through keyword searches or manual extraction by rereading medical reports. To determine covariates influencing PFS, data were extracted as close as possible to the date of the first infusion. Likewise, to determine those influencing the occurrence of grade ≥3 toxicity, data were extracted within 30 days of starting treatment.

### 2.3. Endpoints 

The primary endpoint was treatment response, categorized as follows: (1) complete response (disappearance of disease signs); (2) partial response (disappearance of at least 50% of disease signs); (3) stable disease (no decrease or increase of disease signs); and (4) progression (increased signs of disease). Secondary endpoints were cumulative incidence of grade III or more AE, OS, and PFS. AE were graded according to the National Cancer Institute Common Terminology Criteria for Adverse Events (CTCAE) version 5.0 [40]. CRS and ICANS were graded according to the consensus criteria from the American Society for Transplantation and Cellular Therapies [12].

PFS was measured from the date of first immunotherapy infusion to the date of death from any cause, disease relapse or progression, or the date of data censoring. OS was calculated from the date of first immunotherapy infusion until the date of death from any cause or the date of data censoring.

### 2.4. Statistical Analysis

Quantitative variables were described with means, medians, standard deviation (SD), interquartile range (IQR), and extreme values. Qualitative variables were described with numbers and proportions. OS and PFS were estimated using the Kaplan–Meier method.

A Cox proportional-hazards model with a stepwise selection procedure to select predictive covariates was used to assess the influence of said covariates on PFS as stratified by hematological malignancies (HL vs. DLBCL). DLBCL anti-PD-1 treated patients were excluded from this analysis due to the small sample size. To examine the association between predictive covariates and the occurrence of toxicity grade ≥ 3 within 30 days of the treatment infusion, a logistic regression model was used. This model was stratified according to the type of treatment (CAR T cells vs. anti-PD-1). The toxicities considered were hematological, infectious, digestive, liver, kidney, cardiac, pulmonary, cutaneous, and endocrine for anti-PD-1, with CRS and ICANS additionally considered for CAR T cells.

In order to reduce the amount of missing data, several measures were taken: missing BMI, WBC (White Blood Cells), and hemoglobin values were automatically retrieved from hospitalization and consultation reports when available and plausible, while other variables with missing values were imputed by the MICE algorithm (Multiple Imputation by Chained Equations) with twenty imputations of ten iterations each. The variables to be imputed were as follows: number of previous treatments, BMI, disease stage, PS (performance status), ASAT (aspartate aminotransferase), ALAT (alanine aminotransferase), bicarbonates, bilirubin, creatinine, CRP, GGT (Gamma-glutamyl transferase), hemoglobin, LDH, WBC, alkaline phosphatases, potassium, sodium, and urea, which were imputed based on biological variables, demographics, and comorbidities.

Data management was performed using Python 3.7 (Python software Foundation, Amsterdam, The Netherlands) and quantitative analyses were carried out with R 4.0.0 (R Foundation for Statistical Computing, Vienna, Austria), using the survival package version 3.3 for Cox regression. We followed the recommendations of The STrengthening the Reporting of OBservational Studies in Epidemiology (STROBE) Initiative. A detailed statistical analysis plan was developed and carried out before the data were frozen.

## 3. Results

### 3.1. Demographic and Clinical Characteristics of Patients 

**Figure 1:**
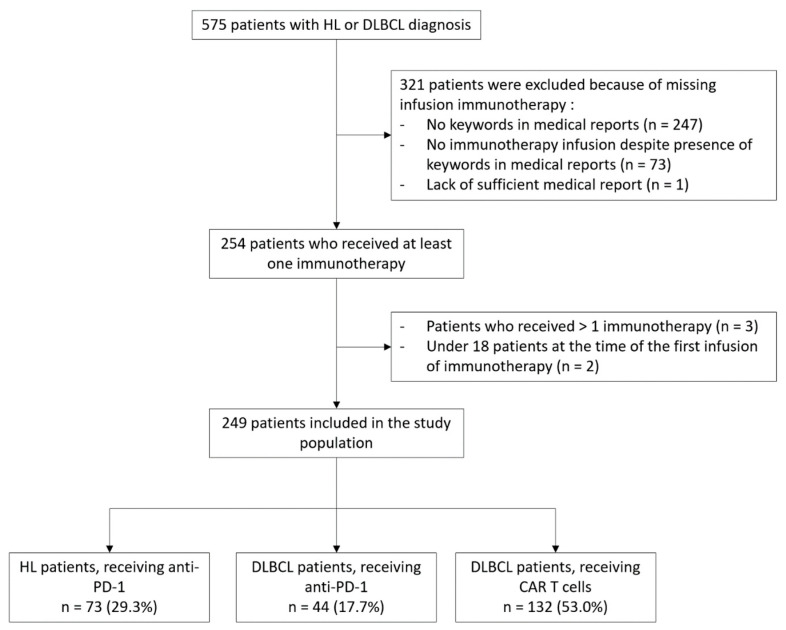
Flow chart of study design and patient inclusion. CAR T cells: chimeric antigen receptor T cells, DLBCL: diffuse large B-cell lymphoma, HL: Hodgkin lymphoma, PD-1: programmed cell death 1.

We retrospectively enrolled 249 lymphoma patients from 21 centres, of whom 117 were treated by anti-PD-1 and 132 by CAR T cells (Figure 1). Seventy-three (29.3%) patients had HL and received anti-PD-1 therapy. One hundred and seventy-six patients (70.7%) had DLBCL; of these, 132 received CAR T cell therapy and the 44 received anti-PD-1 therapy (Table 1).

Among the HL cohort, the patients’ median age at time to treat (TT) was equal to 54.0 years (interquartile range [IQR], 34.0–71.0). Almost two thirds of the patients were men (47/73, 64.4%). Most patients had a PS equal to 0 or 1 (*n* = 41; 69.5%) and a disseminated disease (stage III/IV, *n* = 64; 87.6%). IPS was high in 65.4% (*n* = 17) of patients. Before the first anti-PD-1 infusion, patients received a median of two (IQR, 1–3) prior lines of treatment.

Among the DLBCL cohort treated with anti-PD-1, the patients’ median age at TT was equal to 57.0 years (IQR, 38.5–74.2) and the sex ratio was equal to 1. Twenty-four patients presented a PS < 2 (77.4%) and thirty-nine (88.6%) a disease stage ≥ III. IPI was low in 83.3% (*n* = 15) of patients. Before the first anti-PD-1 infusion, patients received a median of 1.5 (IQR, 1–3) prior lines of treatment.

Among the DLBCL cohort treated by CAR T cells, median age at TT was equal to 63.0 years (IQR, 55.0–69.2). Almost two thirds of the patients were men (82/132, 62.1%). Most patients had a PS equal to 0 or 1 (*n* = 106, 84.1%) and presented a disseminated disease (*n* = 123, 93.2%). IPI was low in 71.1% (*n* = 69) of patients. Before infusion, patients received a median of two (IQR, 2–3) prior lines treatment and 76.5% (*n* = 101) of patients received bridging therapy, including chemotherapy regimens, anti-CD20 containing regimens, or both. Because of lymphoma progression, 5.9% (6/101) received more than one line of bridging therapy. 

### 3.2. Efficacy Outcomes

**Figure 2:**
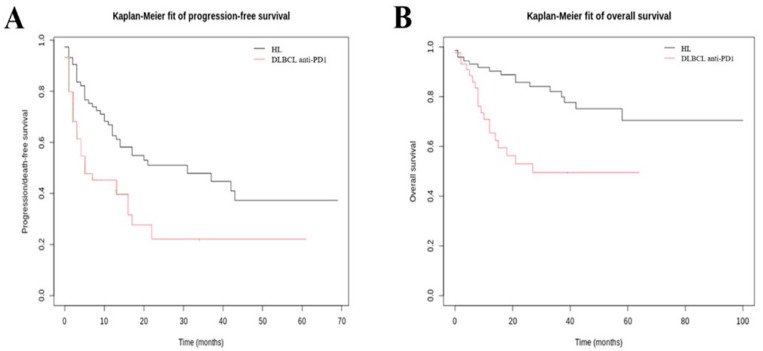
Kaplan–Meier curves of anti-PD-1 treated patients. (**A**) Kaplan–Meier curve for PFS and (**B**) Kaplan–Meier curve for OS. DLBCL: diffuse large B-cell lymphoma; HL: Hodgkin lymphoma; OS: overall survival; PD-1: programmed cell death 1; PFS: progression-free survival.

The best overall response rate (ORR) of HL patients was 51.4%; thirty patients (41.7%) had a complete response, six patients (8.3%) had a partial response, and one patient (1.4%) had a stable disease. Following anti-PD-1 therapy, the median PFS was 16.5 months (IQR, 5.9–29.5) (Figure 2A). Median OS was not reached (Figure 2B).

Among the 44 DLBCL anti-PD-1 treated patients, the best ORR was 34.1%; thirteen patients (29.5%) had a complete response, one patient (2.3%) had a partial response, and one patient (2.3%) had a stable disease. Following anti-PD-1 therapy, the median PFS was 5.3 months (IQR, 2.0–15.5) (Figure 2A). The median OS was 13.3 months (IQR, 7.6–38.6) (Figure 2B).

**Figure 3:**
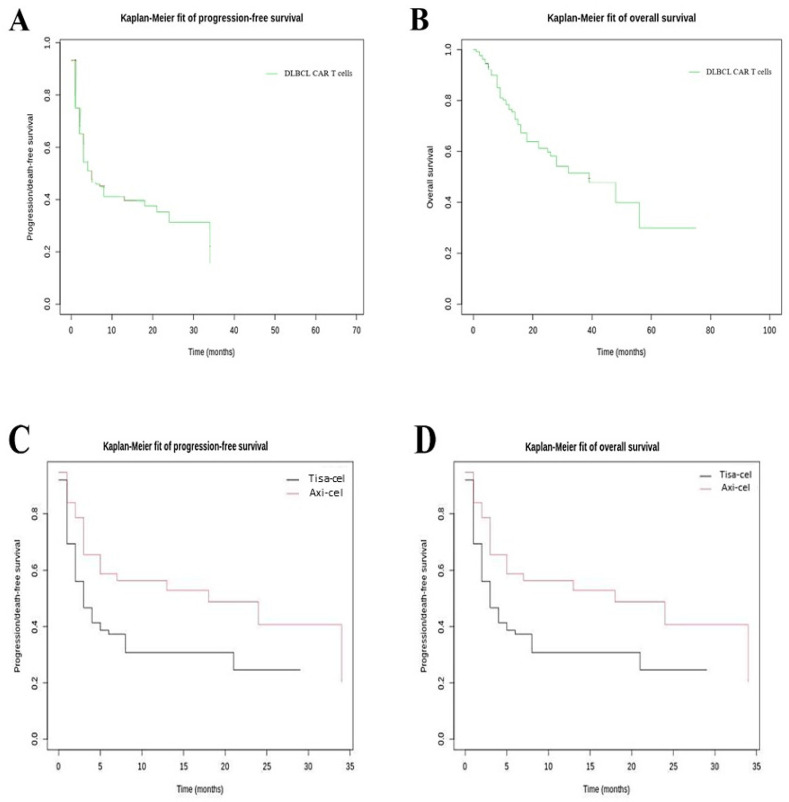
Kaplan–Meier curves of CAR T cell-treated patients. (**A**) Kaplan–Meier curve for PFS, regardless CAR T cells type; (**B**) Kaplan–Meier curve for OS irrespective of CAR T cell type; (**C**) Kaplan–Meier curve for PFS depending on CAR T cell type (tisagenlecleucel versus axicabtagen ciloleucel); and (**D**) Kaplan–Meier curve for OS depending on CAR T cell type (tisagenlecleucel versus axicabtagen ciloleucel). Axi-cel: axicabtagene ciloleucel; CAR T cells: chimeric antigen receptor T cells; DLBCL: diffuse large B-cell lymphoma; OS: overall survival; PFS: progression-free survival; tisa-cel: tisagenlecleucel.

The best ORR of DLBCL CAR T cell-treated patients was 44.3%; 52 patients (39.7%) had a complete response, five patients (3.8%) had a partial response, and one patient (0.8%) had a stable disease. Following CAR T cells, the median PFS was 3.8 months (IQR, 1.6–11.1) (Figure 3A). The median OS from infusion was 15.5 months (IQR, 9.2–26.4) (Figure 3B). The PFS was significantly improved after axi-cel infusion compared to after tisagenlecleucel (tisa-cel) infusion (*p* = 0.008), with a median PFS equal to 5.1 months (IQR, 2.7–14) versus 3.1 months (IQR, 1.0–8.4) (Figure 3C). The median OS was 17.2 months (IQR, 10.2–28.0) and 14.8 months (IQR, 9.1–24.9) for axi-cel and tisa-cel, respectively (Figure 3D). The OS was significantly improved after axi-cel infusion compared to after tisa-cel (*p* = 0.05).

### 3.3. Response Prediction

**Figure 4:**
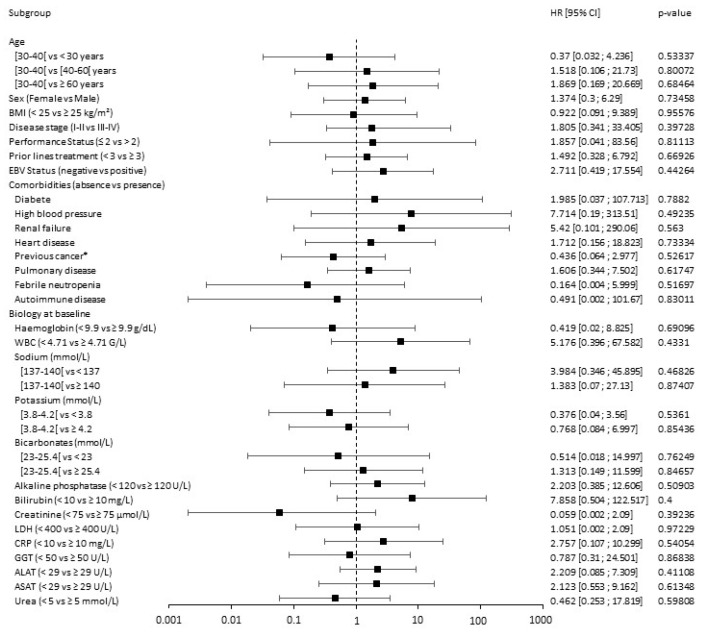
Forest plot of analyses of predictive factors for PFS of HL anti-PD-1 treated patients. ALAT: alanine aminotransferase; ASAT: aspartate aminotransferase; BMI: body mass index; CI: confidence interval; CRP: C-reactive protein; EBV: Epstein–Barr virus; GGT: gamma-glutamyl transferase; HR: hazard ratio; LDH: lactate dehydrogenase; WBC: white blood cells; *: other than hematological. Black points represent the value of the hazard ratio (HR) and segment the value of the 95% CI. The first category in parentheses is taken as the reference category for comparison and HR computation. HR < 1 represents a prognosis factor associated with a prolonged survival or a lower toxicity, while HR > 1 represents a prognosis factor associated with shorter survival or greater toxicity. A prognostic factor is statistically significant if the 95% CI does not contain 1. The Cox univariate model was used for calculating the HR and associated two-sided *p* value.

Univariate analysis of the HL patient cohort is presented in Figure 4. None of age, gender, PS, disease stage, or Epstein–Barr infection influenced PFS in HL patients treated by anti-PD-1 therapy. Moreover, among all clinical assessments considered, none significantly impacted treatment response as measured by PFS.

**Figure 5:**
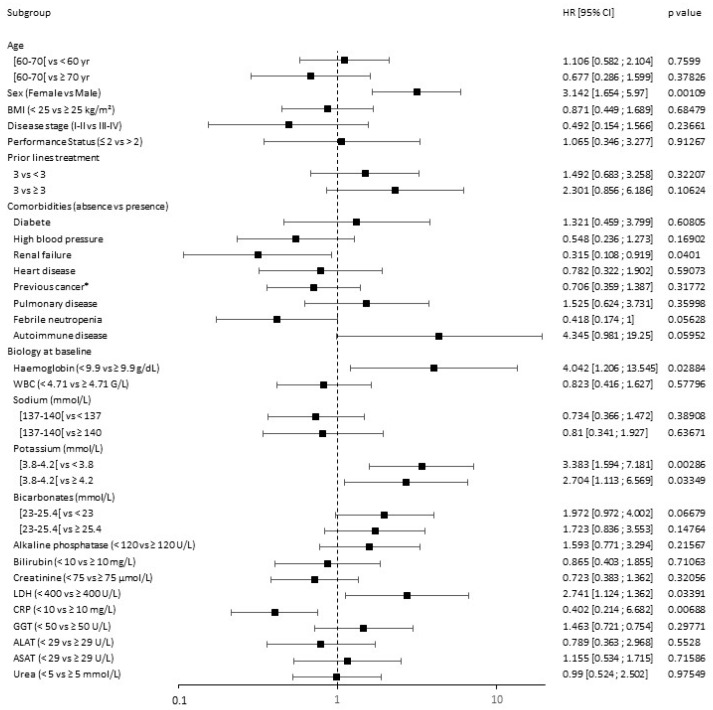
Forest plot of analyses of predictive factors for PFS of DLBCL CAR T cell-treated patients ALAT: alanine aminotransferase; ASAT: aspartate aminotransferase; BMI: body mass index; CI: confidence interval; CRP: C-reactive protein; EBV: Epstein–Barr virus; GGT: gamma-glutamyl transferase; HR: hazard ratio; LDH: lactate dehydrogenase; WBC: white blood cells; *: other than hematological. Black points represent the value of the hazard ratio (HR) and segment the value of the 95% CI. The first category in parentheses is taken as the reference category for comparison and HR computation. HR < 1 represents a prognosis factor associated with a prolonged survival or a lower toxicity, while HR > 1 represents a prognosis factor associated with shorter survival or greater toxicity. A prognostic factor is statistically significant if the 95% CI does not contain 1. The Cox univariate model was used for calculating the HR and associated two-sided *p* value.

The univariate analysis of DLBCL CAR T cell-treated patients is summarized in Figure 5.

Among demographic parameters, male sex was significantly associated with disease progression (HR = 3.142, 95% CI: 1.654–5.97; *p* = 0.00109). Interestingly, age (*p* = 0.7599 for age < 60 and *p* = 0.37826 for age ≥ 70 years old), disease stage (*p* = 0.23661), and PS (*p* = 0.91267) were not predictive factors for PFS. Additionally, early CAR T cell treatment (i.e., < 3 prior lines treatment) compared to later treatment (≥ 3 prior lines treatment) did not impact PFS (*p* = 0.32207 and *p* = 0.10624, respectively). Among comorbidities, there was a trend towards a negative association between autoimmune disorders and PFS (*p* = 0.05952). Among biological factors, LDH ≥ 400 U/L was significantly associated with poorer PFS (HR = 2.741, 95% CI = 1.124–6.682; *p* = 0.03391). Additionally, a rate of haemoglobin strictly inferior to 9.9 g/dL (*p* = 0.02884) and extreme potassium concentration at baseline (i.e., <3.8 and ≥4.2 mmol/L, respectively) were significantly associated with PFS (*p* = 0.00286 and *p* = 0.03349, respectively). 

### 3.4. Adverse Events

The most common anti-PD-1-related AEs (grade superior or equal to 3) for HL and DLBCL patients were blood disorders (78.1% and 59.1%, respectively), hepatic disorders (34.2% and 25.0%, respectively) and kidney disorders (21.9% and 18.2%, respectively) (Table 2). When focusing on patients treated with CAR T cells within 30 days after treatment infusion, 118 out of 132 patients (89.4%) experienced a grade 3 or 4 AE. The most common drug-related AE of grade ≥ 3 were CRS (*n* = 15, 11.4%), ICANS (*n* = 15, 11.4%) and blood disorders (*n* = 40, 30.3%). Overall, 103 patients had a CRS of any grade and 47 patients had an ICANS of any grade (Table 2). 

### 3.5. Toxicity Prediction

**Figure 6:**
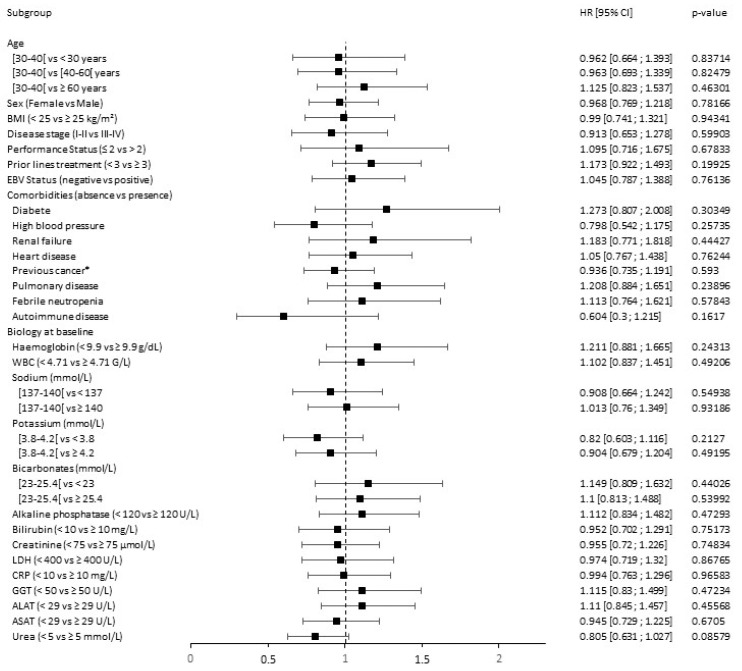
Forest plot of analyses of predictive factors for toxicity of anti-PD-1 treated patients. ALAT: alanine aminotransferase; ASAT: aspartate aminotransferase; BMI: body mass index; CI: confidence interval; CRP: C-reactive protein; EBV: Epstein–Barr virus; GGT: gamma-glutamyl transferase; HR: hazard ratio; LDH: lactate dehydrogenase; WBC: white blood cells; *: other than hematological. Black points represent the value of the hazard ratio (HR) and segment the value of the 95% CI. The first category in parentheses is taken as the reference category for comparison and HR computation. HR < 1 represents a prognosis factor associated with a prolonged survival or a lower toxicity, while HR > 1 represents a prognosis factor associated with shorter survival or greater toxicity. A prognostic factor is statistically significant if the 95% CI does not contain 1. The Cox univariate model was used for calculating the HR and associated two-sided *p* value.

Neither demographic factors, treatment history, or clinical biomarkers influenced the occurrence of toxicity induced by anti-PD-1 (Figure 6).

**Figure 7:**
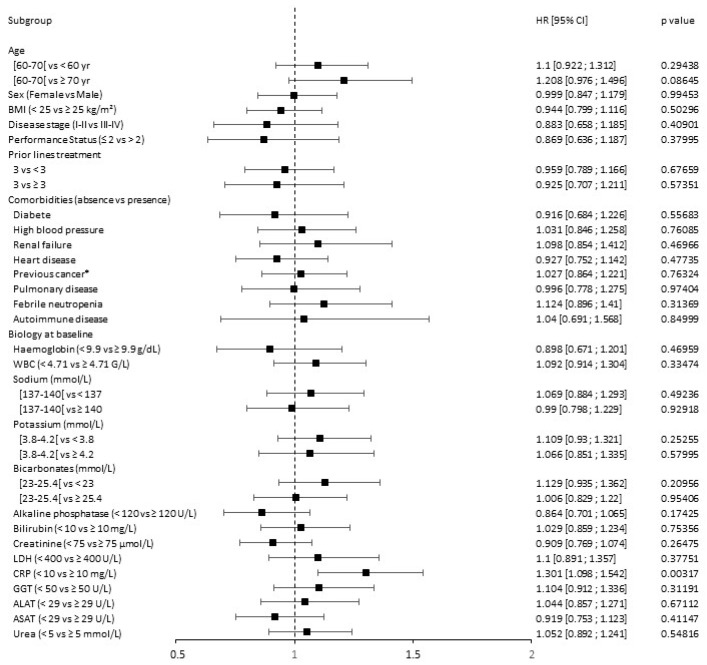
Forest plot of analyses of predictive factors for toxicity of CAR T cell-treated patients. ALAT: alanine aminotransferase; ASAT: aspartate aminotransferase; BMI: body mass index; CI: confidence interval; CRP: C-reactive protein; EBV: Epstein–Barr virus; GGT: gamma-glutamyl transferase; HR: hazard ratio; LDH: lactate dehydrogenase; WBC: white blood cells; *: other than hematological. Black points represent the value of the hazard ratio (HR) and segment the value of the 95% CI. The first category in parentheses is taken as the reference category for comparison and HR computation. HR < 1 represents a prognosis factor associated with a prolonged survival or a lower toxicity, while HR > 1 represents a prognosis factor associated with shorter survival or greater toxicity. A prognostic factor is statistically significant if the 95% CI does not contain 1. The Cox univariate model was used for calculating the HR and associated two-sided *p* value.

Toxicities induced by CAR T cells were not influenced by demographic characteristics, PS, disease stage, treatment history, or comorbidities (Figure 7).

Nevertheless, age ≥ 70 seemed to be negatively associated with CAR T cell tolerance (HZ = 1.208, *p* = 0.08645). Among biological values at baseline, only an elevated CRP portended poorer tolerance to CAR T cells. Indeed, with a hazard ratio equal to 1.301 (95% CI: 1.098–1.542), a CRP level ≥ 10 mg/L was significantly associated with increase in toxicity occurrence (*p* = 0.00317). 

## 4. Discussion

A total of 249 patients with ML treated by anti-PD-1 or CAR T cell therapy were analyzed in this real-world study. Considering survival and toxicity data obtained outside of trials, these findings are consistent with the previous literature. Known predictive response or toxicity factors of CAR T cells such as age, elevated LDH, and CRP at the time of infusion were confirmed by the PRONOSTIM study. Additionally, new factors were highlighted, including gender, hemoglobin, and kalemia.

The median PFS from HL patients treated with anti-PD-1 echoed the ORR of the CHECKMATE 205 and KEYNOTE 087 studies, in which the median PFS was 14.7 and 13.7 and the ORR was 69.0% and 51.4%, respectively [6,7]. Regarding toxicity, the most common drug-related AEs were blood, hepatic, and kidney disorders. 

To the best of our knowledge, most of the published data identifying predictive factors of anti-PD-1 efficacy and toxicity refer to patients with solid tumours, and are not relevant for extrapolation to ML [22,41,42,43,44,45,46]. In our study, the influence of demographic factors, clinical and biological characteristics at baseline, the number of prior lines of treatment, and comorbidities were all considered. We showed the absence of significant influence of age or sex on both PFS and occurrence of adverse events in anti-PD-1 treatment. Sex-based benefit following treatment with anti-PD-1 remains a contentious issue. Although safety profiles and survival of men and women are comparable, differences can be driven by other factors such as age, hormonal status, the influence of oestrogens in women, delay in diagnosis in men, and the greater overall longevity of women [47,48,49,50]. Regarding age, Nishijima et al. and Baldini et al. showed a significant survival benefit with anti-PD-1 in younger patients and in older patients with an age limit of 65–70 years, as well as a more frequent irAE in older subjects [51,52]. None of these studies looked at HL patients. BMI ≥ 25 kg/m^2^ and obesity were not associated with survival or tolerance outcomes; these results are consistent with previous results [16]. Univariate analysis of the above factors did not allow us to identify more significant predictors of survival and toxicity to ICI.

In terms of OS and PFS, our results are consistent with survival rates from the JULIET and ZUMA-1 trials and from the French DESCAR-T registry [1,2,29]. Regarding toxicity, most common adverse events following CAR T cell infusion were CRS and ICANS, occurring in 78.0% (11.4% of grade ≥ 3) and 35.6% (12.1% of grade ≥ 3) of patients, respectively. In the pivotal ZUMA-1 and JULIET studies, grade ≥ 3 CRS was 13.0% for axi-cel and 22.0% for tisa-cel. Similarly, grade ≥ 3 ICANS was 28.0% and 12.0%, respectively. The better safety profile in our real-world study can be explained by better experience over time and new management strategies around CRS and ICANS.

The identification of predictive factors associated with the efficacy and safety of CAR T cell therapy in the DLBCL population is a hot topic. Interestingly, in our study males benefitted less than females from CAR T cells in terms of PFS. To the best of our knowledge this association has not yet been demonstrated, although a previous study highlighted that females were at greater risk of severe toxicity after CAR T cell infusion [53]. As previously demonstrated, no age-related differences in efficacy or toxicity were observed, suggesting that age alone should not limit use of CAR T cells [54,55]. Regarding comorbidities, we tried to use the ICD-10 classification to identify predictive factors. Surprisingly we observed an unexpected positive impact of renal failure on PFS. Outlier results associated with a few patients indicate the existence of a random selection bias and that potential unmeasured confounders may undermine the validity of our conclusions. As previously published, no association between obesity and toxicity or response was identified [56]. Potential markers of greater burden of more aggressive disease have been evaluated in previous studies. Analysis suggests that patients with elevated pre-infusion LDH have poorer PFS [25,57]. LDH greater than 400 U/L levels before CAR T cell infusion was associated with maximal predictive significance for PFS, confirming the cut-off defined by Rabinovich et al. [27]. Literature data remain contentious about the level of LDH and the severity of ICANS or CRS [30,58]. In our study, we did not highlight any correlation between LDH levels and ICANS or CRS severity, in accordance with the results obtained by Pennisi et al. [30]. High CRP levels at baseline have previously been reported in patients with severe CRS or ICANS [30,31]. Our results suggest that a CRP dosage superior or equal to 10 mg/L initially portends poorer tolerance. In another study, CRP levels obtained on the day of CAR T cell infusion strictly inferior to 30 mg/L were correlated with improved duration of response (*p* = 0.003), PFS (*p* < 0.001) and OS (*p* < 0.001), while an increased day 0 and peak CRP were associated with grade ≥ 3 ICANS, though not with CRS [59]. As recently described, moderate toxicity manifesting as grade 2 CRS may be associated with favourable clinical outcomes [60]. Thus, the occurrence of CRS and ICANS was analysed jointly in this study. 

## 5. Conclusions

In this study, we used data from a clinical data warehouse to identify predictors of immunotherapy response in lymphomas. Based on a large set of biological and clinical factors, our results confirm already known predictors and demonstrate male gender, low hemoglobin, and hypo- or hyperkalemia as predictive factors for progression after CAR T cell therapy. These risk factors should be considered together with others already highlighted rather than independently, as evidenced by the emergence of numerous predictive scores. The associations between predictors should be used as an enhanced risk stratification algorithm for decision support before starting treatment, and their usefulness should be confirmed in prospective cohorts. In this context, the use of the clinical data warehouse shows an essential contribution to identifying new predictive characteristics not previously evidenced by clinical trials or more typical real-world studies.

## Figures and Tables

**Table 1:** Baseline demographic and clinical characteristics of patients.

		Hodgkin Lymphoma Cohort	DLBCL Anti-PD-1 Treated Cohort	DLBCL CAR T Cells Treated Cohort
**Characteristics**		*n* = 73	*n* = 44	*n* = 132
**Sex**				
Male	*n* (%)	47 (64.4)	22 (50.0)	82 (62.1)
Female	*n* (%)	26 (35.6)	22 (50.0)	50 (37.9)
**Age at time of (1st) treatment infusion (years)**	Med [IQR]	54.0 [34.0–71.0]	57.0 [38.5–74.2]	63.0 [55.0–69.2]
	min; max	18.0; 86.0	18.0; 84.0	25.0; 80.0
**BMI (kg/m^2^)**	Med [IQR]	23.5 [19.7–25.8]	23.6 [20.3–26.4]	24.5 [21.7–27.1]
	min; max	14.9; 34.7	13.3; 31.6	14.6; 39.5
Including obesity background	*n* (%)	6 (8.2)	1 (2.3)	6 (4.5)
**Comorbidities**				
Diabete	*n* (%)	3 (4.1)	5 (11.4)	11 (8.3)
High blood pressure	*n* (%)	13 (17.8)	6 (13.6)	31 (23.5)
Renal failure	*n* (%)	9 (12.3)	4 (9.1)	15 (11.4)
Heart disease	*n* (%)	18 (24.7)	7 (15.9)	29 (22.0)
Previous cancer *	*n* (%)	18 (24.7)	15 (34.1)	36 (27.3)
Pulmonary disease	*n* (%)	11 (15.1)	7 (15.9)	16 (12.1)
Febrile neutropenia	*n* (%)	6 (8.2)	6 (13.6)	17 (12.9)
Autoimmune disease	*n* (%)	2 (2.7)	1 (2.3)	5 (3.8)
EBV positive status	*n* (%)	18 (24.7)	6 (13.6)	12 (9.1)
**Stage at diagnosis**				
Local disease (I/II stages)	*n* (%)	9 (12.3)	5 (11.4)	9 (6.8)
Dissiminated disease (III/IV stages)	*n* (%)	64 (87.7)	39 (88.6)	123 (93.2)
**PS at diagnosis**				
0–2	*n* (%)	53 (89.8)	27 (87.1)	118 (93.6)
3–4	*n* (%)	6 (10.2)	4 (12.9)	8 (6.4)
Missing	*n* (%)	14 (19.2)	13 (29.5)	6 (4.5)
**Pronostic score at diagnosis**				
IPI				
0–3	*n* (%)	-	15 (83.3%)	69 (71.1%)
≥4	*n* (%)	-	3.0 (16.7%)	28 (28.8%)
Missing	*n* (%)	-	26 (59.1)	35 (26.5)
IPS				
0–3	n (%)	9 (34.6%)	-	-
≥4	n (%)	17 (65.4%)	-	-
Missing	*n* (%)	47 (64.4)	-	-
**Prior lines treatment**	Med [IQR]	2.0 [1.0–3.0]	1.5 [1.0–3.0]	2.0 [2.0–3.0]
	min; max	0.0; 7.0	1.0; 9.0	0.0; 6.0
Including autograft	*n* (%)	16 (21.9)	6 (13.6)	27 (20.5)
**Last PFS (months)**	Med [IQR]	5.0 [2.5–10.0]	5.0 [2.2–10.8]	3.0 [2.0–6.0]
	min; max	1.0; 79.0	1.0; 72.0	1.0; 53.0
Missing	*n* (%)	10 (13.7)	2 (4.5)	1 (0.8)
**Biology at baseline**				
Potassium (mmol/L)	Med [IQR]	4.0 [3.8–4.3]	4.1 [3.8–4.3]	4.1 [3.7–4.3]
	min; max	3.0; 6.3	3.0; 5.1	2.4; 6.4
Missing	*n* (%)	1 (1.4)	7 (15.9)	1 (0.8)
Hemoglobin (g/dL)	Med [IQR]	11.4 [9.8–13.0]	11.4 [9.7–12.8]	10.5 [9.2–12.3]
	min; max	5.0; 16.9	7.7; 15.7	3.3; 15.5
Missing	*n* (%)	0 (0.0)	3 (6.8)	3 (2.3)
LDH (U/L)	Med [IQR]	266.0 [197.0–327.5]	250.5 [202.5–337.5]	334.5 [229.5–544.2]
	min; max	107.0; 2224.0	156.0; 1109.0	134.0; 4428.0
Missing	*n* (%)	2 (2.7)	10 (22.7)	4 (3.0)

BMI: body mass index; CAR T cells: chimeric antigen receptor T cells; DLBCL: diffuse large B-cell lymphoma; HL: Hodgkin lymphoma; IPI: international prognostic index; IPS: international prognostic score; IQR: interquartile range; LDH: lactate dehydrogenase; Med: median; PD-1: programmed cell death 1; PFS: progression-free survival; PS: performance status; * other than hematological.

**Table 2:** Safety analysis of infused patients.

		Hodgkin Lymphoma Cohort	DLBCL Anti-PD-1 Treated Cohort	DLBCL CAR T Cells Treated Cohort
**Adverse events**		*n* = 73	*n* = 44	*n* = 132
**CRS**	*n* (%)	-	-	103 (78.0)
Including grade ≥3	*n* (%)	-	-	15 (11.4)
**ICANS**	*n* (%)	-	-	47 (35.6)
Including grade ≥3	*n* (%)	-	-	16 (12.1)
**Hematological disorders grade ≥3**	*n* (%)	35 (47.9)	16 (36.4)	40 (30.3)
**Hepatic disorders grade ≥3**	*n* (%)	11 (15.1)	4 (9.1)	34 (25.8)
**Kidney disorders grade ≥3**	*n* (%)	5 (6.8)	2 (4.5)	30 (22.7)
**Endocrine disorders grade ≥3**	*n* (%)	7 (9.6)	2 (4.5)	1 (0.8)
**Infections grade ≥3**	*n* (%)	6.0 (8.2)	0.0 (0.0)	35.0 (26.5)
**Cardiac disorders grade ≥3**	*n* (%)	6.0 (8.2)	0.0 (0.0)	9.0 (6.8)
**Skin disorders grade ≥3**	*n* (%)	5 (6.8)	1 (2.3)	3 (2.3)
**Neurological disorders grade ≥3**	*n* (%)	4 (5.5)	1 (2.3)	1 (0.8)
**Digestive disorders grade ≥3**	*n* (%)	3 (4.1)	3 (6.8)	12 (9.1)
**Respiratory and lung disorders grade ≥3**	*n* (%)	3.0 (4.1)	0.0 (0.0)	1.0 (0.8)

CAR T cells: chimeric antigen receptor T cells; CRS: cytokine release syndrome; DLBCL: diffuse large B-cell lymphoma; ICANS: immune effector cell-associated neurotoxicity syndrome; PD-1: programmed cell death 1.

## Data Availability

The datasets generated and/or analyzed during the current study are available from the corresponding author on reasonable request.
